# Prevalence and Characteristics of Sexual Dysfunction in Patients Treated with Immune Checkpoint Inhibitors

**DOI:** 10.3390/medicina61112033

**Published:** 2025-11-14

**Authors:** Betul Aktepe, Oktay Halit Aktepe, Pinar Ezgi Dama, Tugce Ulasli, Ilkay Tugba Unek, Aziz Karaoglu, Mehmet Hamid Boztas, Suayib Yalcin

**Affiliations:** 1 Department of Psychiatry, Urla State Hospital, Ministry of Health, Izmir 35430, Turkey; 2 Department of Medical Oncology, Dokuz Eylul University, Izmir 35330, Turkey; 3 Department of Psychiatry, Bolu Abant Izzet Baysal University, Bolu 14030, Turkey; 4 Department of Medical Oncology, Hacettepe University Cancer Institute, Ankara 06230, Turkey

**Keywords:** ASEX, cancer, GRISS, immune checkpoint inhibitors, sexual dysfunction

## Abstract

*Background and Objectives*: Sexual dysfunction (SD) is common in cancer but remains poorly characterized among patients receiving immune checkpoint inhibitors (ICIs). This study aimed to determine the prevalence and predictors of SD in ICI-treated patients using validated instruments. *Materials and Methods*: In this cross-sectional study, adults with histologically confirmed malignancies who received ≥ 3 cycles of ICIs and reported sexual activity were included. Sexual function was evaluated with the Arizona Sexual Experience Scale (ASEX) and the Golombok–Rust Inventory of Sexual Satisfaction (GRISS). Univariate and multivariate logistic regression analyses identified demographic and clinical predictors of SD. *Results*: Among 208 patients (median age 59 years; 35.1% female), SD prevalence was 66.3% by ASEX and 59.1% by GRISS. ASEX revealed impairment across five domains—sexual drive, psychological and physiological arousal, orgasm, and satisfaction—while GRISS indicated dysfunction mainly in impotence/orgasmic disorder, avoidance, and satisfaction subscales. In multivariate analysis, age ≥ 60 years (OR: 3.14, 95% CI 1.51–6.53, *p* = 0.002), female sex (OR: 3.19, 95% CI 1.31–7.74, *p* = 0.010), Eastern Cooperative Oncology Group (ECOG) performance status ≥ 1 (OR: 2.82, 95% CI 1.39–5.71, *p* = 0.004), ≥2 metastatic sites (OR: 3.08, 95% CI 1.53–6.19, *p* = 0.002), and later treatment lines (OR: 2.43, 95% CI 1.20–4.94, *p* = 0.013) independently predicted ASEX-defined SD. GRISS-based analysis revealed comparable outcomes, identifying ECOG ≥1 and higher metastatic burden as the most prominent predictors of SD, consistent with ASEX findings. *Conclusions*: SD affected nearly two-thirds of patients receiving ICIs. Female sex, later treatment lines, poor ECOG performance status, and higher metastatic burden were key determinants, emphasizing the importance of routine sexual health evaluation in cancer care.

## 1. Introduction

Immune checkpoint inhibitors (ICIs) have transformed the therapeutic paradigm for a range of malignancies, particularly in melanoma, renal cell carcinoma (RCC), and non-small cell lung cancer (NSCLC) [1]. Despite these therapeutic advances, they are also linked to diverse immune-related adverse events (irAEs), ranging from relatively mild skin reactions to life-threatening complications, including those affecting the gastrointestinal tract, endocrine, or nervous systems [2].

Sexual dysfunction (SD) represents a prevalent but underexplored challenge in cancer survivorship. It is a highly common complication, with reported prevalence ranging from 30% to 80%, depending on tumor characteristics, treatment modality, and the instruments used for assessment [3,4,5]. Traditional cancer treatment modalities, including surgery, radiotherapy, chemotherapy, and endocrine treatment, have been shown to contribute to impaired libido, erectile dysfunction, vaginal dryness, orgasmic disorders, arousal difficulties, and relationship strain [6,7,8]. Nevertheless, despite plausible biological and psychosocial explanations, evidence on SD during ICIs remains scarce. Endocrine irAEs, including hypophysitis, hypothyroidism, and adrenal insufficiency, may directly alter gonadal hormone synthesis, leading to reduced libido and SD [9,10]. In addition, patients on ICIs often experience fatigue, mood disturbances, and disrupted sleep, which can further worsen SD [11].

SD can be reliably evaluated using validated tools such as the Arizona Sexual Experience Scale (ASEX) and the Golombok–Rust Inventory of Sexual Satisfaction (GRISS), addressing both individual experiences and relational dynamics [12,13]. Although validated in oncology and chronic disease populations, these instruments have rarely been systematically applied to cancer patients treated with ICIs. Therefore, this study aimed to evaluate the prevalence and characteristics of SD in patients receiving ICIs.

## 2. Materials and Methods

### 2.1. Patient Population

This cross-sectional study enrolled adults (≥18 years) with histologically confirmed malignancies who received treatment with ICIs, including anti–programmed cell death protein 1 (anti–PD-1; nivolumab, pembrolizumab), anti–programmed death-ligand 1 (anti–PD-L1; atezolizumab, durvalumab, avelumab), or anti–cytotoxic T-lymphocyte–associated protein 4 (anti–CTLA-4; ipilimumab) agents at the Departments of Medical Oncology, Bolu Abant İzzet Baysal University and Dokuz Eylül University, between October 2022 and October 2025. Eligibility required completion of at least three cycles of ICIs to ensure adequate treatment exposure, as most immune-related or systemic effects of ICIs typically manifest after the initial few cycles, allowing for a more reliable evaluation of sexual function during active therapy. Additional inclusion criteria were the Eastern Cooperative Oncology Group (ECOG) performance status of 0–2, indicating that patients were ambulatory and able to perform all necessary self-care activities, and self-reported sexual activity within the previous six months to ensure the feasibility of sexual function assessment. Patients were excluded if they had severe cognitive impairment, major psychiatric illness interfering with questionnaire reliability, or neurological disorders known to affect sexual function directly. Further exclusion criteria were pelvic surgery or radiotherapy within the past 12 months, concurrent use of medications with significant sexual side effects (such as anti-androgen therapy or high-dose corticosteroids unless stable for ≥3 months), ECOG performance status ≥ 3, absence of recent sexual activity, or inability to provide informed consent. Demographic, clinical, and treatment-related variables were retrieved from medical records. All participants completed validated self-report questionnaires as part of the standardized study protocol.

### 2.2. Sexual Function Evaluation and Instruments

The ASEX is a brief, validated five-item questionnaire designed to measure core domains of sexual functioning: sexual drive, psychological arousal, physiological arousal (penile erection in men or vaginal lubrication in women), ability to reach orgasm, and satisfaction from orgasm [12]. Each item is scored on a 6-point Likert scale, with higher scores reflecting greater dysfunction. The total score ranges from 5 to 30. A diagnosis of SD was defined according to established cut-off criteria: a total ASEX score ≥ 19, or a score of ≥5 on any single item, or scores of ≥4 on three or more items. The ASEX has been widely used in both clinical and research settings, including cancer populations, because of its brevity, reliability, and sensitivity to changes in sexual function.

The GRISS is a 28-item self-report instrument specifically developed to assess SD in heterosexual couples [13]. Separate male and female versions are available. Items are rated on a five-point scale (always, usually, sometimes, seldom, never), and raw scores are summed and subsequently transformed into standardized sten scores (range: 1–9). Higher sten scores indicate more severe sexual problems. The GRISS provides both a total score and multiple subscales: for men—impotence, premature ejaculation, frequency of sexual intercourse or contact, communication, avoidance, satisfaction, and nonsensuality; for women—orgasmic disorder, vaginismus, frequency of sexual intercourse or contact, communication, avoidance, satisfaction, and nonsensuality. According to conventional interpretation, sten scores of 1–4 are considered normal, five borderline, and 6–9 indicative of clinically relevant dysfunction. The multidimensional structure of GRISS enables detailed exploration of specific domains of sexual relationships, beyond global dysfunction.

### 2.3. Statistical Analysis

Descriptive statistics summarized baseline characteristics. Continuous variables were expressed as mean ± standard deviation (Sd) or median with interquartile range (IQR); categorical variables as frequencies and percentages. Logistic regression models were used to identify predictors of SD. Variables with *p* ≤ 0.20 in univariate analyses were entered into multivariable models. Odds ratios (OR) with 95% confidence intervals (CI) were reported. Spearman’s rank correlation was used to examine the association between total ASEX and GRISS scores. Model calibration was assessed using the Hosmer–Lemeshow goodness-of-fit test. A two-sided *p*-value < 0.05 was considered statistically significant. All analyses were performed using the Statistical Package for the Social Sciences, Version 27.0 (IBM Corp., Armonk, NY, USA).

## 3. Results

### 3.1. Patient Characteristics

A total of 208 patients treated with ICIs were included in the analysis. Demographic and clinical features of patients are presented in Table 1. The median age was 59 years (range, 36–69), and 35.1% were female. The most common primary malignancies were NSCLC (58.7%), RCC (13.9%), and melanoma (10.1%). Other malignancies included colorectal, head and neck, urothelial, gastric, and hepatocellular carcinomas. Most patients received ICIs as second- or third-line therapy (68.3%), whereas 31.7% were treated in the first-line setting. Regarding regimen type, anti–PD-1–based therapy predominated (83.6%), mainly nivolumab (74.0%) and pembrolizumab (9.6%), followed by anti–PD-L1 agents (13.0%), including atezolizumab (8.2%), durvalumab (2.4%), and avelumab (2.4%). A small subset of patients (3.4%) received combination therapy with nivolumab and ipilimumab. The majority of patients (59.1%) had an ECOG performance status of 1–2. The most frequent comorbidities were hypertension (26.4%), diabetes mellitus (8.6%), and cardiovascular disease (12%). The median number of comorbidities was 1 (IQR, 0–2). At the time of sexual function assessment, patients had completed a median of 5 (IQR, 4–7) ICI cycles. The most frequent metastatic site was bone (42.8%), followed by lung (26%), liver (22.1%), and adrenal (12.9%).

### 3.2. Sexual Function Assessment

Sexual function assessment results according to ASEX and GRISS scales are presented in Table 2. Based on ASEX, the mean total score was 19.1 ± 4.9, and 138 patients (66.3%) met the criteria for SD. Across individual ASEX domains, the mean scores were as follows: sexual drive (3.8 ± 1.2), psychological arousal (3.7 ± 1.2), physiological arousal (3.7 ± 1.3), ability to reach orgasm (3.9 ± 1.2), and satisfaction with orgasm (3.8 ± 1.4). Similarly, according to GRISS, the mean total sten score was 6.0 ± 0.7, with 123 patients (59.1%) showing clinically relevant SD. Among the subscales, the most affected domains were impotence/orgasmic disorder (6.6 ± 1.5), avoidance (6.1 ± 1.4), and vaginismus/premature ejaculation (6.1 ± 1.5), followed by satisfaction (5.9 ± 1.5) and frequency (5.8 ± 1.4). Communication was relatively less affected (5.5 ± 1.3). A significant positive correlation was observed between total ASEX and GRISS scores (Spearman’s *r* = 0.578, *p* < 0.001).

### 3.3. Determinants of ASEX- and GRISS-Defined SD

Univariate and multivariate logistic regression analyses were performed to identify clinical and demographic factors associated with SD, as assessed by ASEX (Table 3) and GRISS (Table 4). Both multivariable logistic regression models showed good calibration according to the Hosmer–Lemeshow goodness-of-fit test (ASEX model: χ^2^ = 9.27, *p* = 0.320; GRISS model: χ^2^ = 3.65, *p* = 0.887).

In the ASEX-based analysis, univariate logistic regression showed that age ≥ 60 years, female sex, ECOG ≥ 1, ≥2 metastatic sites, and second-/third-line therapy were significantly associated with ASEX-defined SD. After adjustment in the multivariate model, age ≥ 60 years (OR: 3.14, 95% CI 1.51–6.53, *p* = 0.002), female sex (OR: 3.19, 95% CI 1.31–7.74, *p* = 0.010), ECOG ≥ 1 (OR: 2.82, 95% CI 1.39–5.71, *p* = 0.004), ≥2 metastatic sites (OR: 3.08, 95% CI 1.53–6.19, *p* = 0.002), and second-/third-line therapy (OR: 2.43, 95% CI 1.20–4.94, *p* = 0.013) remained independent predictors. For the GRISS-based analysis, univariate logistic regression indicated that age ≥ 60 years (OR: 2.42, *p* = 0.003), female sex (OR: 3.25, *p* = 0.002), ECOG ≥ 1 (OR: 4.65, *p* < 0.001), ≥2 metastatic sites (OR: 4.19, *p* < 0.001), ≥median treatment cycles (OR: 1.83, *p* = 0.035), and second-/third-line therapy (OR: 4.62, *p* < 0.001) were all significantly associated with GRISS-defined SD. In the multivariate model, female sex (OR: 3.07, 95% CI 1.32–7.13, *p* = 0.009), ECOG ≥ 1 (OR: 4.48, 95% CI 2.17–9.24, *p* < 0.001), ≥2 metastatic sites (OR: 4.38, 95% CI 2.14–8.96, *p* < 0.001), and second-/third-line therapy (OR: 2.98, 95% CI 1.45–6.10, *p* = 0.003) remained significant predictors.

## 4. Discussion

Our results demonstrate that SD is a common but often overlooked issue in ICI-treated cancer patients. These validated tools provided a detailed assessment, encompassing overall sexual health as well as individual domains. In this multicenter cross-sectional study, we observed that SD was prevalent in nearly two-thirds of ICI-treated patients, indicating that ICIs—despite their survival benefit—have a measurable impact on intimate functioning and quality of life.

These results are in line with emerging evidence suggesting that ICIs can influence sexual health through biological and psychosocial pathways. Previous studies have predominantly focused on endocrine irAEs such as hypophysitis, hypothyroidism, and adrenal insufficiency, which disrupt gonadal hormone synthesis and reduce libido [9,14]. Additionally, inflammatory cytokine elevations during ICI treatment—particularly interleukin-6 and tumor necrosis factor-alpha—may interfere with neuroendocrine signaling, mood regulation, and sleep quality [15,16], all of which are known contributors to SD. Notably, dysfunction in orgasm-related domains was the most pronounced across both ASEX and GRISS subscales in our cohort, which may be partly explained by these hormonal and neuroinflammatory alterations, as well as potential ICI-induced neuropathic or autonomic effects that can impair orgasmic response. Taken together, these biological alterations, compounded by fatigue and psychosocial distress that frequently accompany ICI therapy [17], may collectively contribute to impaired sexual well-being in this population.

From a clinical perspective, these findings carry important implications. The observation that nearly two-thirds of patients experienced SD during ICI therapy highlights sexual health as an essential yet frequently neglected aspect of survivorship care. ICIs have increasingly provided durable survival benefits across a wide range of tumor types [18,19]. These advances have also introduced chronic toxicities affecting psychosocial domains. In both ASEX- and GRISS-based analyses, female sex, ECOG ≥1, and multiple metastatic sites emerged as independent predictors, reflecting the complex interplay between biological vulnerability, disease burden, and psychosocial stressors.

The higher prevalence of SD in later treatment lines may reflect the cumulative effects of prior therapies and ongoing fatigue or emotional strain. Nevertheless, this issue remains relevant across all treatment lines, as immunotherapy use expands into earlier settings, where survivorship considerations and long-term quality of life will become increasingly critical. Future longitudinal studies should examine temporal dynamics of SD during and after ICIs, including hormonal recovery, endocrine irAEs, and patient-reported outcomes. To our knowledge, this is the first study to apply both ASEX and GRISS systematically in an ICI-treated population, thereby providing a multidimensional characterization of sexual health in the context of immunotherapy.

Clinically, management of SD in immuno-oncology requires a multidisciplinary approach involving oncology, endocrinology, and psycho-oncology specialists. Beyond endocrine irAE monitoring, routine assessment of fatigue, mood alterations, and psychosocial distress is essential to provide comprehensive supportive care for cancer patients receiving ICIs. Supportive care interventions such as psychological counseling, sexual therapy, and partner-focused programs may alleviate symptom burden and contribute to improved quality of life [20,21,22]. Enhancing awareness among oncologists and patients may help overcome barriers and foster open discussion of sexual health concerns. Ultimately, incorporating sexual health into survivorship care is essential to optimize quality of life and maintain therapeutic adherence in cancer patients treated with ICIs.

This study has limitations. The sample size was modest, and only patients who had received at least three cycles of immunotherapy were included, with assessments performed at a median of three months after treatment initiation. Thus, our findings may not reflect longer-term changes in sexual health or outcomes in patients who discontinue treatment earlier due to progression or toxicity. Furthermore, the lack of long-term follow-up data limits our understanding of how sexual function evolves throughout and after ICIs. Whether SD improves, persists, or worsens over time remains uncertain. Future longitudinal studies with serial assessments are warranted to confirm these findings and to delineate the temporal dynamics and potential reversibility of SD associated with ICIs. Additionally, as our data were derived from two tertiary oncology centers within the same country, the generalizability of our findings may be limited. Another limitation is that metabolic comorbidities such as hypertension, diabetes mellitus, and cardiovascular disease were not included in the regression models. However, only patients with well-controlled or stable forms of these conditions were enrolled, minimizing their potential confounding influence. Finally, the lack of hormonal or biochemical data limits mechanistic interpretation, and future studies should incorporate endocrine evaluation to better clarify the biological pathways underlying SD.

## 5. Conclusions

In this study, approximately two-thirds of patients treated with ICIs experienced clinically significant SD as assessed by validated instruments, underscoring the need to integrate sexual health evaluation into survivorship care for patients receiving ICIs. Routine use of standardized tools such as ASEX and GRISS may facilitate early recognition and management.

## Figures and Tables

**Table 1:** Demographic and clinical features of patients treated with ICIs.

Variable	*n* (%)
Age, years (median, range)	59 (36–69)
Sex Male Female	135 (64.9%) 73 (35.1%)
Primary cancer type Non–small cell lung cancer Renal cell carcinoma Melanoma Others	122 (58.7%) 29 (13.9%) 21 (10.1%) 39 (17.3%)
Line of immunotherapy First-line Second-line Third-line or beyond	66 (31.7%) 129 (62%) 13 (6.3%)
Regimen type Anti–PD-1 Anti–PD-L1 Anti–CTLA-4/combination	174 (83.6%) 27 (13%) 7 (3.4%)
ECOG performance status 0 1–2	85 (40.9%) 123 (59.1%)
Comorbidities Hypertension Diabetes mellitus Cardiovascular disease	55 (26.4%) 18 (8.6%) 25 (12%)
Number of comorbidities, median	1 (0–2)
Metastatic sites Lung Liver Bone Adrenal	54 (26%) 46 (22.1%) 89 (42.8%) 27 (12.9%)
ICI cycle at sexual assessment	5 (4–7)

Abbreviations: CTLA-4: cytotoxic T-lymphocyte–associated protein 4; ECOG: Eastern Cooperative Oncology Group; ICI: immune checkpoint inhibitor; PD-1: programmed cell death protein 1; PD-L1: programmed death-ligand 1.

**Table 2:** Sexual function assessment according to ASEX and GRISS scales.

Parameter	Mean ± Sd	Patients Meeting SD Criteria, *n* (%)
ASEX total score (<19 = normal)	19.1 ± 4.9	138 (66.3%)
Sexual drive	3.8 ± 1.2	
psychological arousal	3.7 ± 1.2	
Physiological arousal	3.7 ± 1.3	
Orgasm	3.9 ± 1.2	
Satisfaction	3.8 ± 1.4	
GRISS total sten score (1–4 = normal)	6 ± 0.7	123 (59.1%)
GRISS subscales (mean sten ± SD)		
Avoidance	6.1 ± 1.4	
Satisfaction	5.9 ± 1.5	
Communication	5.5 ± 1.3	
Frequency	5.8 ± 1.4	
Impotence/Orgasmic disorder	6.6 ± 1.5	
Premature ejaculation/Vaginismus	6.1 ± 1.5	

Abbreviations: ASEX: Arizona Sexual Experience Scale; GRISS: Golombok–Rust Inventory of Sexual Satisfaction; Sd: standard deviation; SD: sexual dysfunction.

**Table 3:** Univariate and multivariate logistic regression analyses for factors associated with SD (ASEX-defined).

Variable	Univariate OR (95% CI)	*p*	Multivariate OR (95% CI)	*p*
Age ≥ 60 years	4.01 (2.09–7.70)	<0.001	3.14 (1.51–6.53)	0.002
Female sex	3.28 (1.49–7.19)	0.003	3.19 (1.31–7.74)	0.010
ECOG ≥ 1	3.64 (1.99–6.65)	<0.001	2.82 (1.39–5.71)	0.004
≥2 metastatic sites	3.48 (1.90–6.37)	<0.001	3.08 (1.53–6.19)	0.002
≥median ICI cycles	1.78 (0.99–3.19)	0.051	1.12 (0.56–2.24)	0.734
Second-/third-line therapy	3.81 (2.05–7.08)	<0.001	2.43 (1.20–4.94)	0.013

Abbreviations: CI: confidence interval; ECOG: Eastern Cooperative Oncology Group; OR: odds ratio; SD: sexual dysfunction.

**Table 4:** Univariate and multivariate logistic regression analyses for factors associated with SD (GRISS-defined).

Variable	Univariate OR (95% CI)	*p*	Multivariate OR (95% CI)	*p*
Age ≥ 60 years	2.42 (1.34–4.35)	0.003	1.52 (0.75–3.09)	0.242
Female sex	3.25 (1.56–6.78)	0.002	3.07 (1.32–7.13)	0.009
ECOG ≥ 1	4.65 (2.55–8.45)	<0.001	4.48 (2.17–9.24)	<0.001
≥2 metastatic sites	4.19 (2.31–7.59)	<0.001	4.38 (2.14–8.96)	<0.001
≥median ICI cycles	1.83 (1.04–3.22)	0.035	1.14 (0.57–2.28)	0.699
Second-/third-line therapy	4.62 (2.48–8.61)	<0.001	2.98 (1.45–6.10)	0.003

Abbreviations: CI: confidence interval; ECOG: Eastern Cooperative Oncology Group; GRISS: Golombok–Rust Inventory of Sexual Satisfaction; OR: odds ratio; SD: sexual dysfunction.

## Data Availability

The data presented in this study are available on request from the corresponding author due to privacy and ethical restrictions related to patient confidentiality.

## Abbreviations

The following abbreviations are used in this manuscript:

ASEX	Arizona Sexual Experience Scale
CI	Confidence interval
CTLA-4	Cytotoxic T-lymphocyte–associated protein 4
ECOG	Eastern Cooperative Oncology Group
GRISS	Golombok–Rust Inventory of Sexual Satisfaction
ICI	Immune checkpoint inhibitor
IQR	Interquartile range
irAE	Immune-related adverse event
NSCLC	Non–small cell lung cancer
OR	Odds ratio
PD-1	Programmed cell death protein 1
PD-L1	Programmed death-ligand 1
RCC	Renal cell carcinoma
SD	Sexual dysfunction
Sd	Standard deviation
